# Altered Gut Microbiota in Irritable Bowel Syndrome and Its Association with Food Components

**DOI:** 10.3390/jpm11010035

**Published:** 2021-01-08

**Authors:** Zahra A. Barandouzi, Joochul Lee, Kendra Maas, Angela R. Starkweather, Xiaomei S. Cong

**Affiliations:** 1 School of Nursing, Emory University, Atlanta, GA 30322, USA; zahra.barandouzi@emory.edu; 2 School of Nursing, University of Connecticut, Storrs, CT 06269, USA; angela.starkweather@uconn.edu; 3 Department of Statistics, University of Connecticut, Storrs, CT 06269, USA; joochul.lee@uconn.edu; 4 Microbial Analysis, Resources, and Services (MARS), University of Connecticut, Storrs, CT 06269, USA; kendra.maas@uconn.edu; 5 Department of Pediatrics, School of Medicine, University of Connecticut, Farmington, CT 06106, USA

**Keywords:** irritable bowel syndrome, microbiota, microbiome, food components, nutrients

## Abstract

The interplay between diet and gut microbiota has gained interest as a potential contributor in pathophysiology of irritable bowel syndrome (IBS). The purpose of this study was to compare food components and gut microbiota patterns between IBS patients and healthy controls (HC) as well as to explore the associations of food components and microbiota profiles. A cross-sectional study was conducted with 80 young adults with IBS and 21 HC recruited. The food frequency questionnaire was used to measure food components. Fecal samples were collected and profiled by 16S rRNA Illumina sequencing. Food components were similar in both IBS and HC groups, except in caffeine consumption. Higher alpha diversity indices and altered gut microbiota were observed in IBS compared to the HC. A negative correlation existed between total observed species and caffeine intake in the HC, and a positive correlation between alpha diversity indices and dietary fiber in the IBS group. Higher alpha diversity and gut microbiota alteration were found in IBS people who consumed caffeine more than 400 mg/d. Moreover, high microbial diversity and alteration of gut microbiota composition in IBS people with high caffeine consumption may be a clue toward the effects of caffeine on the gut microbiome pattern, which warrants further study.

## 1. Introduction

Irritable bowel syndrome (IBS) is a chronic functional gastrointestinal disorder with an estimated prevalence of 10% around the globe [1]. This common functional disorder has significant impacts on patients’ quality of life as well as increases enormous economic burdens of on healthcare systems [1,2]. IBS patients suffer from various ranges of symptoms, including abdominal pain/discomfort, abdominal bloating, and alteration in the bowel habits [3]. While the pathophysiology of IBS is not well understood, the interplay between diet and the gut microbiota has gained interest in recent years [4].

Diet is one of the known triggers and/or exacerbators of IBS symptoms [5]. Up to 70% of IBS patients associate their symptoms to specific foods such as dairy products, caffeine, raw vegetables, beans, peas, hot spices, fried foods, alcohol, fatty foods, as well as wheat products [3,6,7]. Although individuals may have selective food choices, dietary patterns, intake of calories, proteins, carbohydrates, and fats by patients with IBS is comparable to community controls [6].

The microbial composition in patients with IBS has been reported to be different from healthy individuals, despite the fact that their dietary patterns were found similar [6]. Studies show lower microbial diversity as well as a decrease in abundance of *Ruminococcaceae, Bifidobacterium*, *Faecalibacterium*, and *Erysipelotrichaceae* in IBS patients compared to healthy individuals. In addition, a higher abundance of *Lactobacillus* and *Ruminococcus* was reported in IBS patients [8,9]. Although evidence supports that IBS patients have altered gut microbiota profiles, it is still largely unknown about the microbial signature that can characterize these patients and their symptoms [6].

Diet as an important environmental factor has a strong impact on the gut microbiota enterotypes [5,6]. Diet enriched in protein and animal fat is associated with the *Bacteroides* enterotype, whereas a diet enriched in carbohydrate is related to the *Prevotella* enterotype [10]. Research also shows that the gut microbiota that belongs to the Firmicutes and Bacteriodetes phyla have an imperative role in the metabolism of carbohydrates and proteins by producing health-beneficial short-chain fatty acid (SCFAs) [11,12]. SCFAs are essential to fuel the intestinal epithelial cells and strengthen the gut barrier function [12]. In recent years, the interplay between diet and microbiota has emerged as an important pathological basis for IBS, which requires further investigation [4,13,14]. Moreover, the role of caffeine consumption on microbiome composition has been evaluated in different diseases, but limited studies have assessed the impact of caffeine in the IBS population [15,16]. Thus, in the present study, we aimed to assess the differences in nutrient intake and gut microbiota patterns between IBS and healthy control (HC) groups; meanwhile, we explored the associations between gut microbial community and food components in both IBS and HC groups.

## 2. Materials and Methods

### 2.1. Setting and Subjects

The present study was an extension of a randomized clinical trial titled “Precision Pain Self-Management in Young Adults with IBS” (P20 NR016605-01) [17]. In this trial, 80 people with IBS diagnosed by a gastroenterologist were enrolled in a longitudinal study. We used the data from the baseline session of this clinical trial and also recruited 21 healthy participants in the study. A convenience sampling method was used in the parent randomized controlled trial (RCT) and recruitment of healthy controls. A retrospective post hoc power analysis was conducted using the G-power program to examine if the sample size reached enough power to detect the effect IBS group on the alpha diversity compared to HC group. The powers of 0.86 for the total observed species (sobs) and 0.91 for Shannon index were obtained when assuming Laplace distribution of the parent response variables.

The inclusion criteria for the enrollment of IBS people were: (1) Men and women 18–29 years of age, (2) with a diagnosis of IBS from a healthcare provider using the Rome III or IV criteria, and (3) able to read and speak in English. The exclusion criteria were: (1) Having other chronic painful conditions including but not limited to fibromyalgia, chronic pelvic pain or chronic intestinal cystitis, infectious diseases (hepatitis, HIV, MRSA), celiac disease or inflammatory bowel disease, and diabetes mellitus, (2) serious mental health conditions (e.g., bipolar disorder, schizophrenia, mania), (3) women who were pregnant or post-partum 3 months, or (4) regular use of opioids, iron supplements, prebiotics/probiotics or antibiotics, and/or substance abuse. The criteria for recruitment of HC were the same as those for the IBS group, except that HC group did not have a history of IBS. The study was approved by the Institutional Review Board of the University of Connecticut. The information of the research study was explained to the participants, and all the participants provided written informed consent.

### 2.2. Data Collection

Both IBS and HC groups completed demographic and food frequency questionnaires via a Research Electronic Data Capture (REDCap) software/system. After receiving explicit instructions from a research team member, the participants were requested to collect their fecal samples using the OMNIgene GUT tubes (DNA Genotek Inc., Ottawa, ON, Canada) and delivered the sample to the lab via a drop-box. The fecal samples were aliquoted into bead tubes and were stored in a −80 °C freezer until further analysis.

### 2.3. Outcome Meaures

#### 2.3.1. Assessment of Daily Food Components

The food frequency questionnaire (FFQ) [18] was used to assess the participants’ dietary patterns. The questionnaire contained questions indicating the frequency of various types of foods, e.g., bread and savory biscuits, cereals, potatoes, rice and pasta, meat and fish, dairy products and fats, sweets and snacks, drinks, soups, sauces and spreads, fruits, and vegetables. The FFQ data was processed using Diet*Calc software developed by the National Institutes of Health National Cancer Institute [19] to obtain data of nutrient and food group intake. The estimation of daily food components based on 24-h dietary recall were calculated according to the portion size for participants’ food energy (kcal), protein (g), total fat (g), cholesterol (mg), carbohydrate (g), dietary fiber (g), alcohol (g), and caffeine (mg).

#### 2.3.2. Fecal Sample DNA Extraction and Microbiome Sequencing

The fecal sample processing, sequencing, and analysis were conducted at the University Center of Microbial Analysis, Resources, and Services using the protocols developed and tested by our team [20,21]. The bacterial DNA were extracted from 0.25 g of the fecal sample using the MoBio Power Soil or PowerMag Soil DNA isolation kit (MoBio Laboratories, Inc, Carlsbad, CA, USA) in accordance with the manufacturer’s instruction for the Eppendorf epMotion 5076 Vac liquid handling robot or manually. Then, the V4 region of the 16S rRNA gene of the microbial community was sequenced using the Illumina platform. For the microbiome analysis, we used the Mothur software. Alpha diversity, including sobs, Simpson, and Shannon indices were used to evaluate the complexity of the whole microbial community. Beta diversity represented by Bray–Curtis dissimilarity was used to indicate the inter-subjects’ variation in the bacterial composition. Multidimensional scaling (MDS) based on Bray–Curtis dissimilarity was used to identify the microbial clustering patterns and assess the relationships with food component intakes.

### 2.4. Statistical Analysis

The demographic characteristics of the participants were presented with frequency and percentage for categorical variables, and mean, standard deviation, and range for continuous variables. A chi-square test and Fisher’s exact test were conducted to check the association between the demographic characteristics and the groups, and the Wilcoxon rank-sum test was conducted to investigate differences of age, number of household members, and daily food component intakes between the IBS and HC groups using R 3.6.0. For analysis of microbiota composition, we dropped operational taxonomic unit (OTUs) in which a ratio of zero counts was identified in more than 90% of the samples, and performed the linear discriminant analysis effect size (LEfSe) method provided at https://huttenhower.sph.harvard.edu/galaxy. An alpha level for the Kruskal–Wallis test and a threshold for the effect size were 0.05 and 2, respectively. To compare the alpha diversity between the IBS and HC groups, a Wilcoxon rank-sum test was used and the propensity score weighting method was further used to control confounding variables in weighed regression models. The Kruskal–Wallis test was utilized to identify differences in alpha diversity among groups and Spearman’s rho correlation to examine the association between the alpha diversity and daily caffeine and dietary fiber intake. Lastly, based on Bray–Curtis dissimilarity, non-metric multidimensional scaling (NMDS) ordination for beta diversity was performed, and we fitted environmental variables related to food components onto the ordination to investigate the association between the beta diversity and food components using the ‘vegan’ package in R.

## 3. Results

### 3.1. Demographic Characteristics of the IBS and HC Groups

In total, 80 individuals with IBS and 21 HC were included in the study. There were no significant differences of age, gender, race, ethnicity, education, caregiver type, employment status, marital status, and number of household members between the IBS and healthy control groups (Table 1).

### 3.2. Food Componnets in the IBS and HC Groups

**Figure 1:**
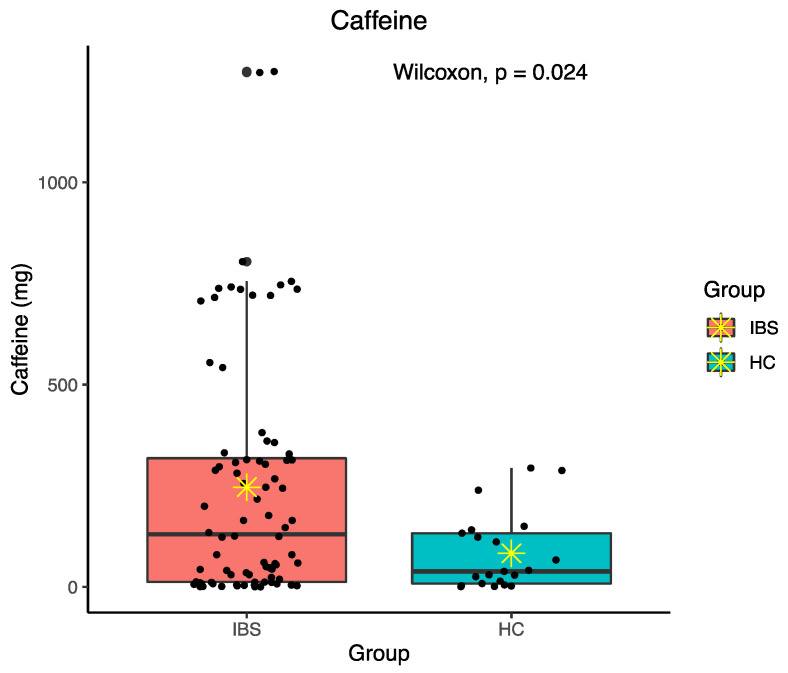
Difference in daily caffeine intake between irritable bowel syndrome (IBS) and healthy control (HC) groups; ✴ indicates mean.

Daily food component intakes were calculated for food energy, protein, fat, cholesterol, carbohydrate, dietary fiber, alcohol, and caffeine. There was no significant difference in daily intakes of various food components between the IBS and HC groups except in caffeine consumption (*p* = 0.024) (Figure 1). The IBS group had higher daily caffeine intake with an average of 246.42 mg/d. The details of daily food components intakes in both groups is shown in Table 2.

### 3.3. Fecal Microbiota Pattern in the IBS and HC Groups

#### 3.3.1. Total Number of OTUs

A total of 483,740 OTUs were identified and analyzed in the study. Respectively, 381,900 OTUs belonged to the IBS group and 101,840 OTUs belonged to the HC group.

#### 3.3.2. Fecal Microbiota Composition in the IBS Compared to the HC

**Figure 2:**
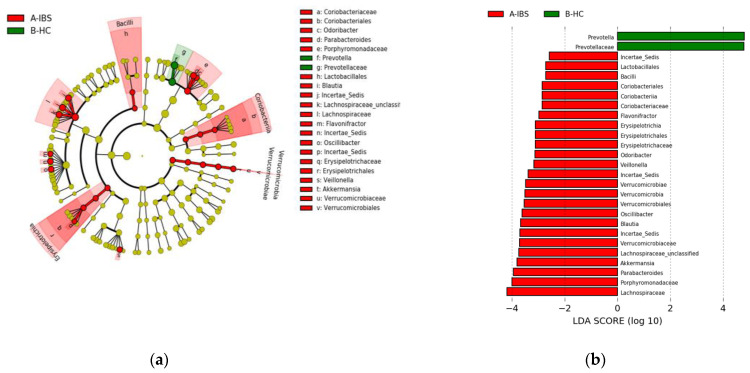
Taxonomic differences of fecal microbiota between IBS and HC groups. (**a**) Taxonomic cladogram based on the linear discriminant analysis effect size (LEfSe) analysis. (**b**) IBS-enriched taxa are indicated with a negative linear discriminant analysis (LDA) score (red) and taxa enriched in HC have a positive score (green).

**Figure 3:**
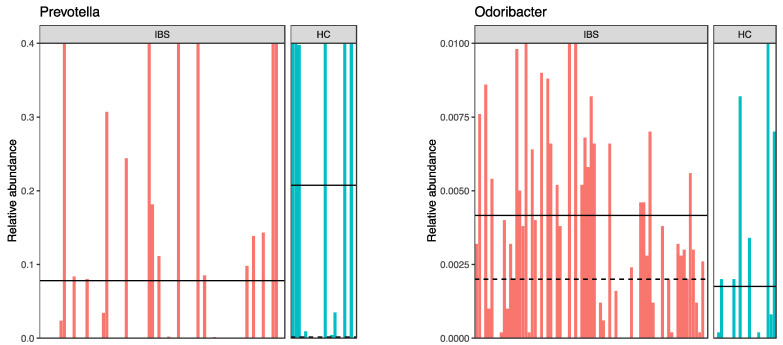

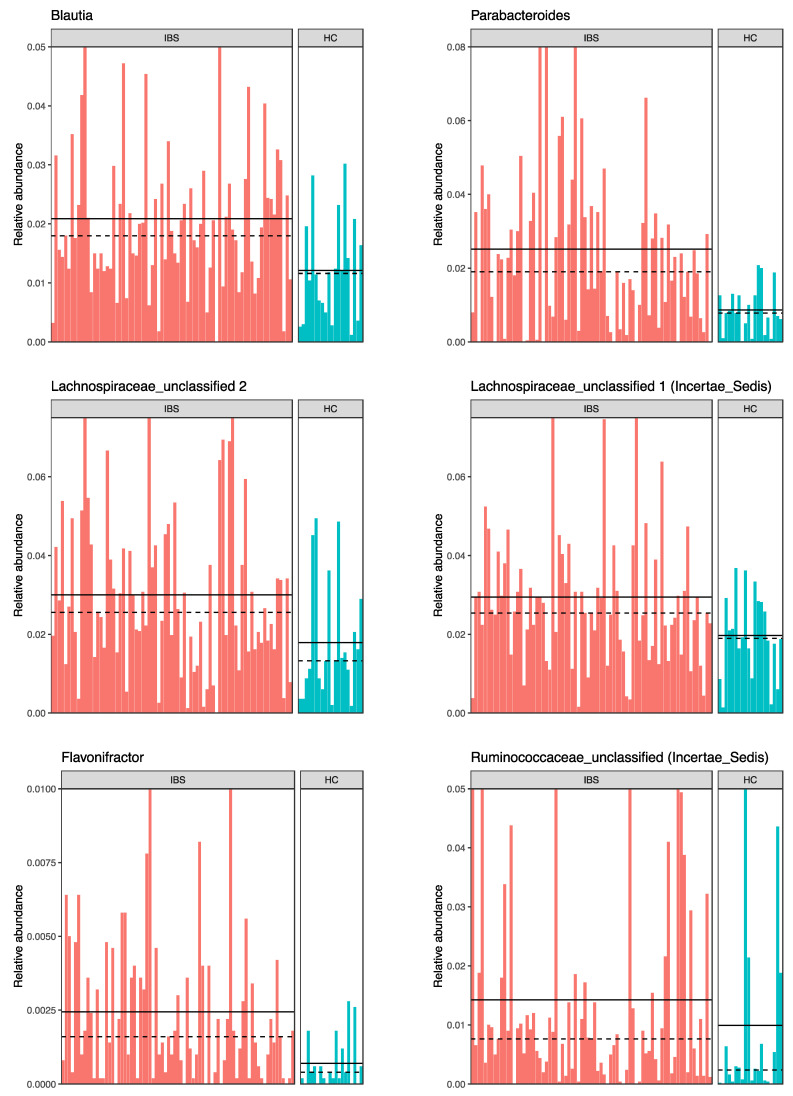

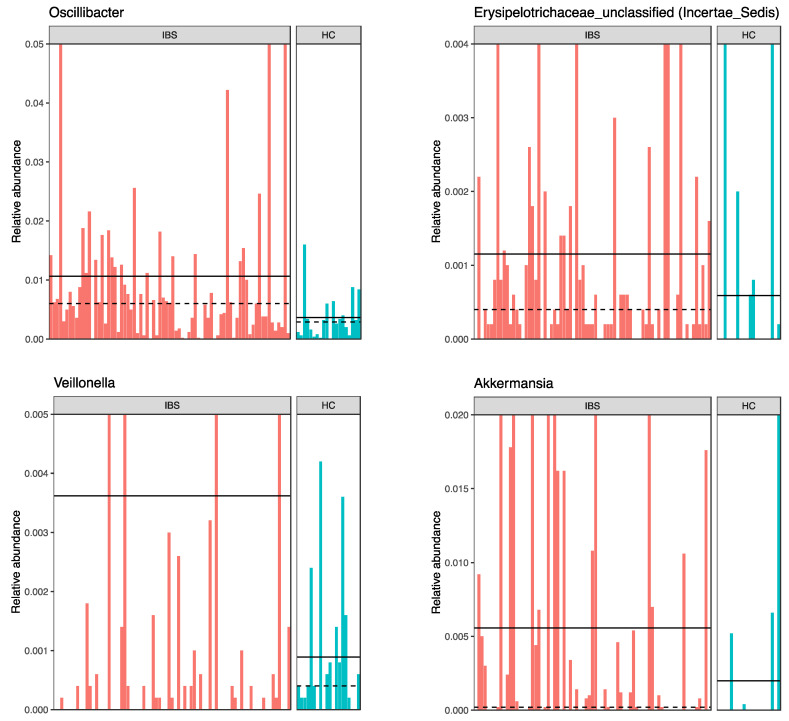
Relative abundance of bacterial genera in each subject of the IBS and HC groups.

The linear discriminant analysis effect size (LEfSe) was utilized to identify the key phylotype responsible for the differences between the IBS and HC groups (Figure 2 and Figure 3). At the phylum level, the IBS group exhibited significantly higher abundance of *Verrucomicrobia* phylum compared to the HC group. At the class level, *Verrucomicrobia, Coriobacteriia, Bacilli*, and *Erysipelotrichia* were more abundant in the IBS group than the HC group. At the order level, we observed higher abundance of *Verrucomicrobiales, Coriobacteriales, Lactobacillales*, and *Erysipelotrichales* in the IBS group compared to the HC group. At the family level, there was higher abundance of *Coriobacteriaceae, Porphyromonadaceae, Verrucomicrobiaceae, Lachnospiraceae,* and *Erysipelotrichaceae* in the IBS group, while a higher abundance of *Prevotellaceae* was observed in the HC group. Among various genera, *Parabacteroides, Blautia, Lachnospiraceae-*unclassified 1*, Lachnospiraceae-*unclassified 2*, Veillonella, Oscillibacter, Flavonifractor, Ruminococcaceae-*unclassified*, Odoribacter, Erysipelotrichaceae-*unclassified*,* and *Akkermansia* were relatively more abundant in the IBS group compared to the HC. However, the abundance of *Prevotella* was more abundant in the HC group compared to the IBS group (Figure 2 and Figure 3).

#### 3.3.3. Fecal Microbiota Diversity in the IBS Compared to the HC Group

**Figure 4:**
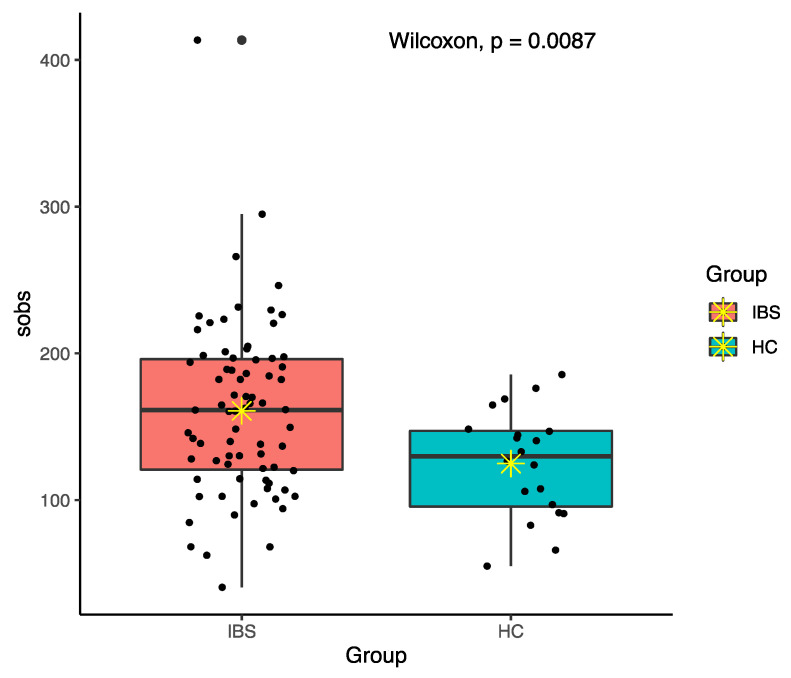
Total observed species (sobs) diversity between IBS and HC groups; ✴ indicates mean.

**Figure 5:**
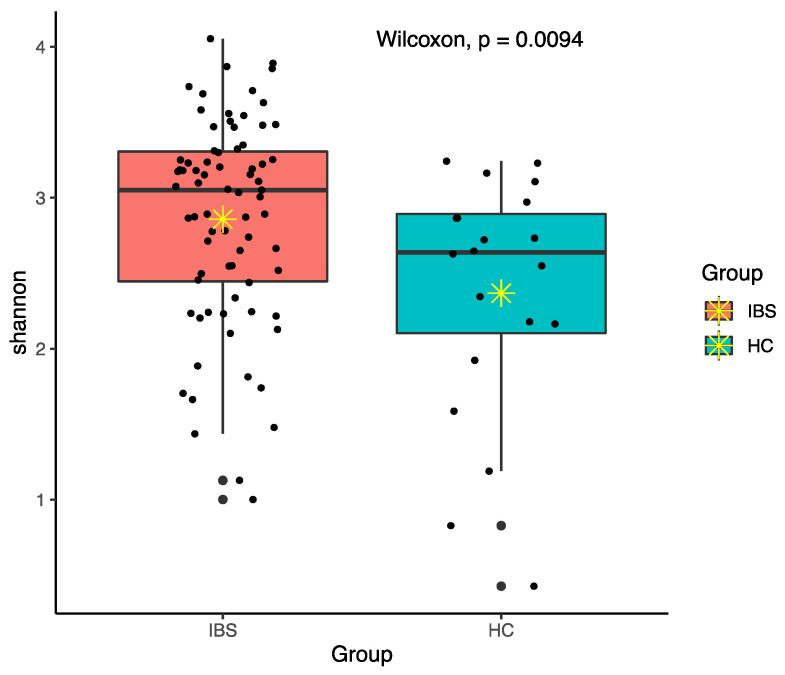
Shannon diversity between IBS and HC groups; ✴ indicates mean.

Among various alpha diversity indices, total observed species (sobs) and the Shannon index were significantly higher in the IBS group compared to the HC group (Figure 4 and Figure 5). However, beta-diversity using the Bray–Curtis index was not structurally different between the two groups. In order to reduce the confounding effects of demographics and food intakes on gut microbiome between the IBS and HC groups, we further applied propensity score weighting methods to give weights to all subjects and run a weighted regression model. The results consistently showed significant difference in alpha diversity indices (sobs: β = 0.188, t = 2.374, *p* = 0.020; Shannon: β = 1.918, t = 2.539, *p* = 0.013).

### 3.4. Associations between Fecal Microbiota Diversity and Food Component Intakes

**Figure 6:**
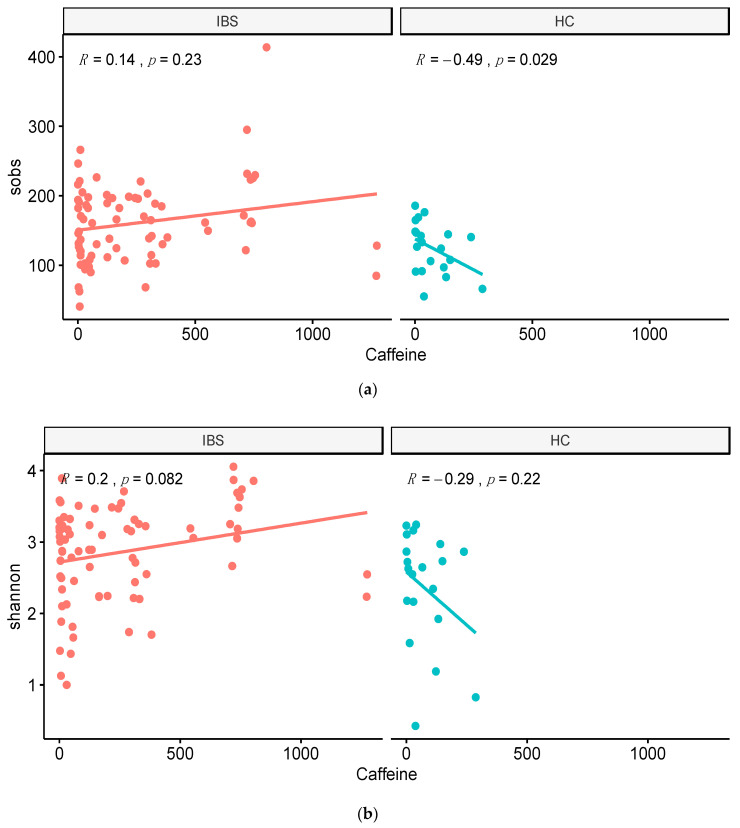
Correlation between caffeine intake and alpha diversity in the IBS and HC groups. (**a**) Correlation between total observed species (sobs) and caffeine intake. (**b**) Correlation between Shannon index and caffeine intake.

**Figure 7:**
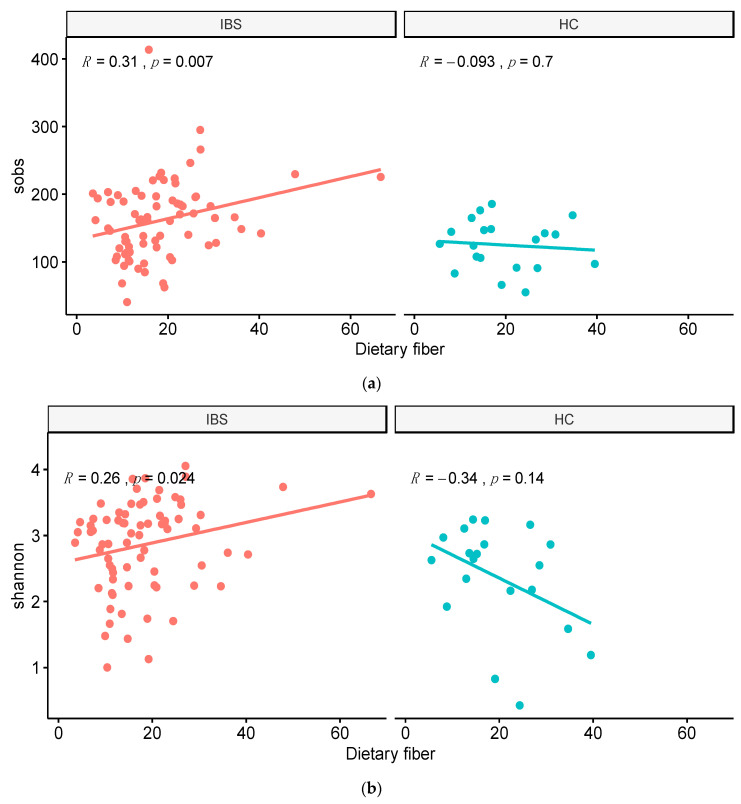
Correlation between dietary fiber and alpha diversity in the IBS and HC groups. (**a**) Correlation between total observed species (sobs) and dietary fiber intake. (**b**) Correlation between Shannon index and dietary fiber intake.

Among different nutrient intakes, we observed a significant correlation between caffeine intake and sobs in the HC group (Figure 6). Moreover, the dietary fiber intake was significantly associated with alpha diversity indices including sobs and the Shannon index in the IBS group (Figure 7).

### 3.5. Fecal Microbiota Patterns Associated with the Daily Caffeine Intake

Due to the high daily consumption of caffeine in the IBS group, we further explored the impact of caffeine intake on the fecal microbiota composition and diversity. Thus, we divided the IBS group into two subgroups including High-IBS and Low-IBS. High-IBS refers to IBS people who consumed caffeine more than 400 mg/day and Low-IBS indicating IBS subjects with less than 400 mg/day caffeine consumption. This caffeine consumption cut-off was based on the US Food and Drug Administration (FDA) recommendation [22,23].

**Figure 8:**
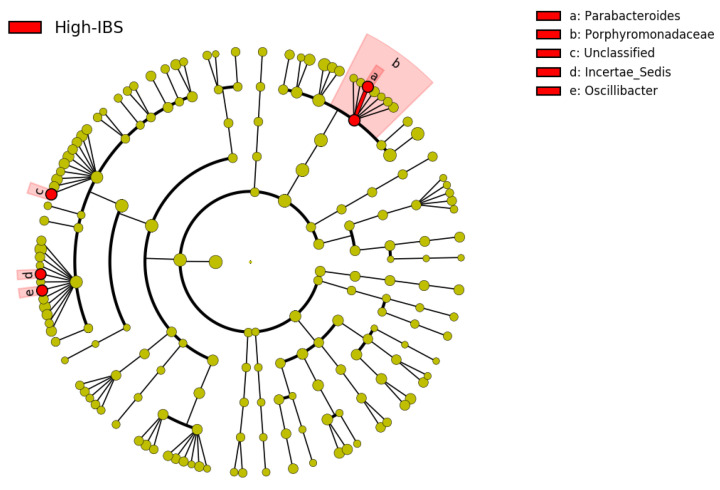
Taxonomic cladogram based on the LEfSe analysis. Red: Taxa enriched in High-IBS; High-IBS: Caffeine consumption more than 400 mg/day.

**Figure 9:**
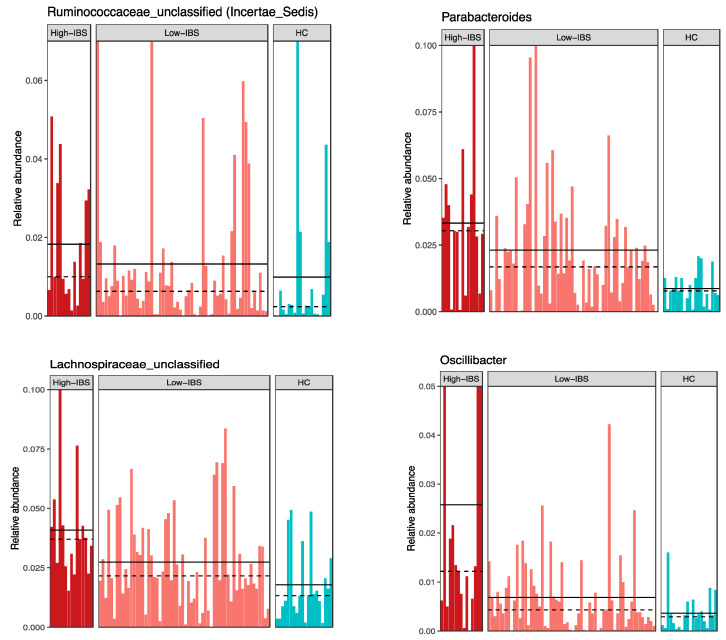
Abundance of bacterial genera in High-IBS, Low-IBS, and HC groups. High-IBS: Caffeine consumption more than 400 mg/day; Low-IBS: Caffeine consumption less than 400 mg/day.

Various genera were more abundant in the High-IBS group compared to the Low-IBS and also the HC groups. Among different genera*, Parabacteroides, Lachnospiraceae*-unclassified, *Ruminococcaceae*-unclassified, and *Oscillibacter* had high relative abundance in IBS people with high consumption of caffeine (Figure 8 and Figure 9).

**Figure 10:**
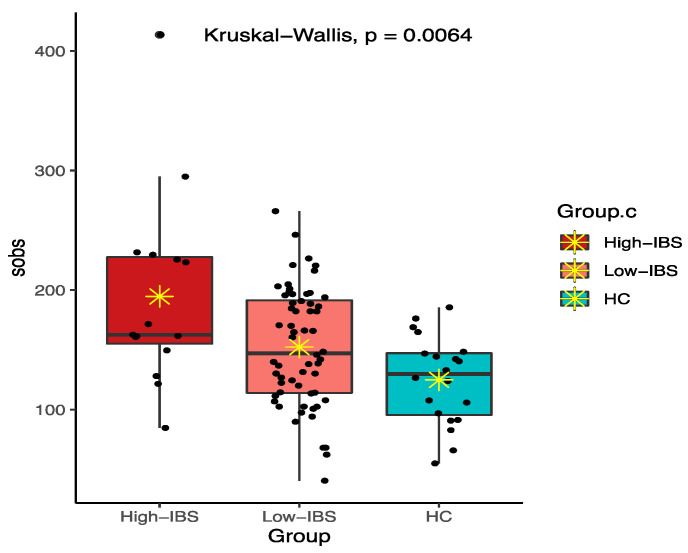
Total observed species (sobs) diversity among High-IBS, Low-IBS and HC groups. High-IBS: Caffeine consumption more than 400 mg/day; Low-IBS: Caffeine consumption less than 400 mg/day; ✴ indicates mean.

**Figure 11:**
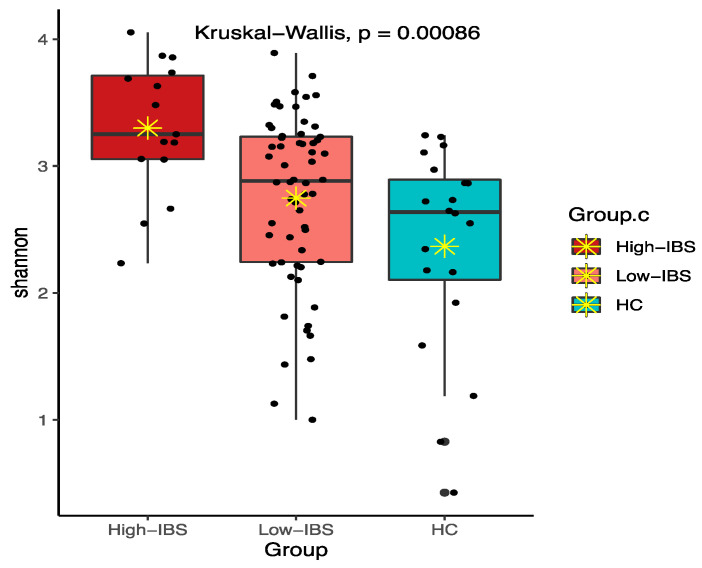
Shannon diversity among High-IBS, Low-IBS and HC groups. High-IBS: Caffeine consumption more than 400 mg/day; Low-IBS: Caffeine consumption less than 400 mg/day; ✴ indicates mean.

The High-IBS group also had a higher alpha diversity profile compared to the Low-IBS and HC groups using the sobs and Shannon indices. Interestingly, the bacterial diversity was higher in the Low-IBS group compared to the HC (Figure 10 and Figure 11).

### 3.6. Associations between Fecal Microbiota Diversity and Food Component Intakes in the High-IBS, Low-IBS, and HC Groups

In terms of the association between bacterial diversity and food component intakes, we observed a negative correlation between sobs and caffeine intake within the HC group. However, there was no significant correlation between alpha diversity (sobs and Shannon index) and caffeine intake in both High-IBS and Low-IBS groups (Supplementary Materials Figure S1). Similarly, we did not identify any correlation between alpha diversity and dietary fiber intake in all three groups (Figure S2). No significant associations were identified among beta diversity and various food components including caffeine and dietary fiber intakes among High-IBS, Low-IBS, and HC groups (Figure S3).

## 4. Discussion

The results of the present study revealed that among various food components, caffeine intake was significantly different between IBS participants and the healthy controls in young adults. Moreover, the microbiome diversity and composition of IBS people were distinct from healthy controls. Correlation analysis of diet and microbiome showed a significant association between caffeine intakes with alpha diversity. Moreover, microbiome diversity was higher in the IBS group who consumed caffeine more than 400 mg/day compared to the IBS low caffeine consumption and HC groups.

### 4.1. Differences in Food Components between IBS and HC

The pathophysiology of IBS from the nutritional aspect is multifaceted and unsettled. IBS is a term to describe various presentations of a dysfunction and no single process can be determined as its pathophysiology [24]. There is no evidence to propose that people who developed IBS in the past had a significantly distinctive diet from healthy people [24]. This study supports evidence from previous research which shows that the intake of main food components such as carbohydrate, calories, proteins, and fats in people who currently suffer from IBS is similar to healthy adults [25,26]. While people with IBS traits may report certain food items associated with their symptoms, the overall food intake pattern is comparable to a healthy community [6].

The results of the present study showed higher daily consumption of caffeine in IBS people compared to healthy controls. In contrast to our findings, one study reported similar caffeine intake with a mean of 1.7 servings/day in people with IBS and healthy controls [27]. Caffeine’s role as a trigger of IBS symptoms is unknown, but reducing its intake is recommended to improve reflux symptoms in people with IBS [6]. In addition, research shows that caffeine influences gut motility in healthy people. However, its role in people with IBS is not clear, which requires further investigations [27,28].

Current IBS dietary guidelines mainly focus on increasing dietary fiber and reduction of fat, caffeine, and alcohol intakes [29,30]. The theory behind the dietary restriction is that caffeine, high-fat food content, and alcohol may play roles in triggering IBS symptoms, and dietary fiber can help to reduce symptoms [13]. While the association between dietary restriction and IBS symptoms has been reported in studies, data regarding the manipulation of a dietary plan in IBS people are still inconsistent [30,31].

### 4.2. Differences in the Gut Microbiota between IBS and HC

The microbiota is an extremely diverse and metabolically active community that can play an imperative role in health and disease [32]. The host–microbiota interactions as a mutualistic ecosystem is beneficial for both host and microbiota [32,33]. A growing body of evidence is proposing gut microbiota dysbiosis as potential pathogenesis of IBS [1].

Two major phyla, *Firmicutes* and *Bacteroidetes*, constitute around 90% of the known bacteria in the gastrointestinal (GI) tract [34]. While we did not see any difference in the abundance of these phyla between IBS people and healthy controls, other studies reported contradictory results. One study found a higher abundance of *Bacteroidetes* and a lower abundance of *Firmicutes* in IBS people [35]. However, other studies reported lower abundance of *Bacteroidetes* in people with IBS [11,36]. Among other phyla, we observed a high abundance of *Verrucomicrobia*. Other studies also reported an elevated abundance of *Proteobacteria* and *Actinobacteria* in IBS people [36,37]. The variation in abundance of different phyla in IBS may be a clue toward alteration of gut microbiota that influences IBS symptoms.

Research suggests that healthy people harbor three types of enterotypes, including *Bacteroides*, *Prevotella,* and *Ruminococcus* [38]. Consistently, our results showed a higher abundance of *Prevotella* in healthy people. However, one study reported high abundance of *Ruminococcus* in people with IBS [11]. Among other genera, we observed higher abundance of *Blautia* in IBS people. Similarly, another study found a higher abundance of this genus in people with IBS [11]. Previous studies revealed *Blautia* and its belonged family, *Lachnospiraceae*, as a potential marker of imbalance in the gut, are associated with numerous diseases [39,40,41,42]. Reports of various genera in different studies suggest a large inter-individual variability in microbiota composition, which requires further studies [34].

Multiple reports have linked IBS pathogenesis with either decreased or unchanged of microbial diversity and richness [9]. Most of the studies reported lower sobs, Chao 1, and Shannon diversities in people with IBS [35,36,43]. In addition, some studies showed no distinction in terms of microbiome diversity between IBS patients and healthy people [44,45]. In contrast to the earlier findings, we observed higher sobs and Shannon diversities in IBS people compared to healthy controls. Supporting this, a study found higher microbiome diversity in IBS patients compared to healthy controls [46]. A possible explanation for these contradictory findings might be related to various techniques of DNA sequencing in different regions for specifying the diversity of the gut microbiota in studies as well as difference in the IBS population [47]. Thus, further studies using similar methodologies are required to help to distinguish IBS people from healthy ones via gut microbiota diversity.

### 4.3. Correlations between Food Components and the Gut Microbiota in IBS

Diet and it’s macro/micronutrient components may influence the gut microbiome either directly or indirectly [48]. The majority of the recent studies have focused on the effects of low fermentable oligo-, di-, and monosaccharides and polyols (FODMAPs) diet in IBS. A low-FODMAP diet has been linked to a reduced abundance of Bifidobacteria, with potential health benefits still under debate [49]. Other studies have reported lower bacterial abundance following the introduction of a low-FODMAP diet compared with a habitual diet [50,51].

While in the low-FODMAP diet, consumption of fermentable and short-chain carbohydrate is restricted, adequate intakes of fiber is encouraged [52]. Dietary fiber has soluble and insoluble components. Though the insoluble fiber is utilized less by the gut microbiota, the soluble components of dietary fiber such as inulin and fructans are mostly used by the gut microbiota as an energy source and help to develop some beneficial bacteria, such as *Lactobacillus* and *Bifidobacteria* [53]. Research also shows the enrichment of the genus *Prevotella* in individuals with higher fiber diets [31]. *Prevotella* is a genus with a high abundance in healthy people. Thus, dietary fiber may help to develop beneficial microbiota in the human gut.

In the current study, we observed a positive correlation between dietary fiber intake and microbial diversity in people with IBS. Higher diversity and richness of the microbiota has also been shown in Agrarian vs. Western diet style communities [31,53]. Fermentation of dietary fiber by microbial fulfills some beneficial influence by production of metabolites. One of the metabolites is short-chain fatty acids (SCFA), which can reduce colonic pH and inhibits the growth of pathogens [54]. Butyrate, as another metabolite provides energy substrate to enterocytes and some bacterial species and enhances the expression of some epithelial tight junction proteins [11,54]. More research is required to determine the role of specific gut microbiota in the fermentation of fiber and the specific metabolites produced.

Our results revealed higher bacterial diversity as well as a higher abundance of some genus, including *Parabacteroides, Oscillibacter, Lachnospiraceae-*unclassified*,* and *Ruminococcaceae-*unclassified in the IBS group who consumed caffeine more than 400 mg/d compared to the HC. Studies on the role of caffeine consumption on microbial diversity and composition are limited. In one study, regular consumption of coffee more than 45 mL/day was associated with a higher level of *Prevotella, Bacteroides,* and *Porphyromonas* in healthy individuals [55]. In another study using a spontaneous mouse model of metabolic syndrome, daily intake of coffee or its components for 16 weeks changed the abundance of various genera such as *Coprococcus, Blautia,* and *Prevotella* in mice [56]. Caffeine, as the major water-soluble component of coffee, influences gut microbiota diversity and patterns. However, its role in the alteration of the gut microbiota remains unclear and requires further investigation [56,57].

Our study may have several limitations that need to be considered when interpreting the results. The study population was narrowed to young adults with IBS and HC. The majority of the young adults recruited in the study were students and their lifestyle may affect their diet and eventually, their gut microbiota patterns. Moreover, using Rome III or IV criteria for recruitment of IBS people might make our study population heterogonous. The small sample size may affect the generalization of the results. Further studies with a larger sample size and more homogenous population by considering the history of diet and medication use are recommended to determine the interplay between diet and microbiota in IBS symptoms.

## 5. Conclusions

In summary, our result revealed similar nutrient intake patterns between IBS people and HC groups except in the daily consumption of caffeine. The gut microbiome communities were significantly different between the IBS and HC groups in terms of microbial diversity and compositions. Higher caffeine consumption in the IBS group was also associated with higher bacterial diversity as well as an alteration in microbial composition. Taken together, these results suggest the influence of caffeine on gut microbiota patterns. Further studies are necessary to investigate the interplay between caffeine intake and gut microbiota.

## Figures and Tables

**Table 1:** Demographic characteristics of the participants.

Demographics	N	HC (*n* = 21)		IBS (*n* = 80)	*p*-Value
Gender					
Female	72	11 (52.38%)		61 (76.25%)	0.060
Male	29	10 (47.619%)		19 (23.75%)	
Race					
White	71	9 (42.86%)		62 (77.50%)	0.070
Asian	16	6 (28.57%)		10 (12.50%)	
African–American	12	4 (19.05%)		8 (10.00%)	
Not reported	2	2 (9.52%)		0 (0.00%)	
Ethnicity					
Non-Hispanic	84	16 (76.19%)		68 (85.00%)	0.360
Hispanic	11	4 (19.05%)		7 (8.75%)	
Not reported	6	1 (4.76%)		5 (6.25%)	
Education					
High school or lower	8	2 (9.52%)		6 (7.50%)	0.151
Some college	63	16 (76.19%)		47 (58.75%)	
Associate degree	3	1 (4.76%)		1 (1.25%)	
Bachelor degree	16	2 (9.52%)		14 (17.50%)	
Master degree	12	0 (0.00%)		12 (15.00%)	
Primary caregiver					
Parent/legal guardian	53	14 (66.67%)		39 (48.75%)	0.117
Self	46	6 (28.57%)		40 (50.00%)	
Other	2	1 (4.76%)		1 (1.25%)	
Employment status					0.269
Student	75	18 (85.71%)		57 (71.25%)	
Working now	22	2 (9.52%)		20 (25.00%)	
Unemployed	4	1 (4.76%)		3 (3.75%)	
Marital status					
Never married	98	21 (100.00%)		77 (96.25%)	1
Married	3	0 (0.00%)		3 (3.75%)	
IBS subtype					
IBS-C	9	N/A		9 (11.00%)	
IBS-D	5	N/A		5 (7.00%)	
IBS-M	66	N/A		66 (82.00%)	
Medical care setting type					
Primary	15	N/A		15 (19.00%)	
Secondary	6	N/A		6 (7.00%)	
Primary + secondary	22	N/A		22 (28.00%)	
None	37	N/A		37 (46.00%)	
	**Mean (SD)**		**Range**		
	**HC**	**IBS**	**IBS**	**HC**	***p*-value**
Age (years)	20.14 (1.39)	20.39 (2.57)	18–23	18–28	0.071
Household members	4.19 (1.81)	3.29 (1.48)	1–9	1–7	0.034
Duration of IBS (years)	N/A	4.01 (2.67)	1–13	N/A	N/A

IBS-C, IBS constipation, IBS-D, IBS diarrhea, IBS-M, IBS-mixed (constipation + diarrhea), N/A, Not applicable.

**Table 2:** Daily food component intakes.

	Mean (SD)		Median (Range)	
	HC	IBS	HC	IBS
Food energy (kcal)	1965.82 (791.06)	1793.41 (761.95)	1837.46 (705.94–3768.59)	1692.51 (320.12–4223.59)
Protein (g)	83.31 (37.13)	73.29 (39.40)	83.05 (21.84–147.22)	60.48 (10.61–216.97)
Fat (g)	87.80 (41.55)	77.46 (36.56)	96.45 (21.81–182.24)	73.40 (9.90–176.68)
Cholesterol (mg)	278.76 (84.29)	228.06 (123.41)	291.71 (52.14–507.28)	205.45 (9.02–543.25)
Carbohydrate (g)	211.73 (84.29)	201.33 (84.90)	194.76 (86.60–398.42)	186.39 (27.94–453.60)
Dietary fiber (g)	19.79 (9.06)	18.44 (10.03)	16.97 (5.55–39.52)	17.31 (3.54–66.61)
Alcohol (g)	4.99 (4.56)	5.56 (5.19)	4.06 (0.01–15.60)	4.30 (0.00–19.16)
Caffeine (mg) *	82.93 (94.67)	246.42 (297.42)	38.24 (0.55–293.77)	129.92 (0.06–1273.84)

* Significant difference in median of caffeine intake (*p* < 0.05).

## Data Availability

The data presented in this study are available on request from the corresponding author. The data are not publicly available due to ongoing data analysis.

## Supplementary Materials

The following are available online at https://www.mdpi.com/2075-4426/11/1/35/s1, Figure S1: Correlation between caffeine intake and alpha diversity. (1) Correlation between total observed species (sobs) and caffeine intake. (2) Correlation between Shannon index and caffeine intake. High-IBS: Caffeine consumption more than 400 mg/day; Low-IBS: Caffeine consumption less than 400 mg/day. Figure S2: Correlation between dietary fiber intake and alpha diversity. (A) Correlation between total observed species (sobs) and dietary fiber intake. (B) Correlation between total Shannon index and dietary fiber intake. High-IBS: Caffeine consumption more than 400 mg/day; Low-IBS: Caffeine consumption less than 400 mg/day. Figure S3: Correlation between beta diversity and nutrient intakes in High-IBS, Low-IBS, and HC groups. High-IBS: Caffeine consumption more than 400 mg/day; Low-IBS: Caffeine consumption less than 400 mg/day; Caff: Caffeine; Fiber: Fiber; and HC groups: High-IBS: Caffeine consumption more than 400 mg/day; Low-IBS: Caffeine consumption less than 400 mg/day; Caff: caffeine; Fiber: Fiber.
