# Supplementary material for: Altered Gut Microbiota in Irritable Bowel Syndrome and Its Association with Food Components
Source: J Pers Med. 2021 Jan 8;11(1):35. doi: 10.3390/jpm11010035 (PMC7827153; doi:10.3390/jpm11010035)
Supplement: Supplementary file 1 [file jpm-11-00035-s001.pdf]

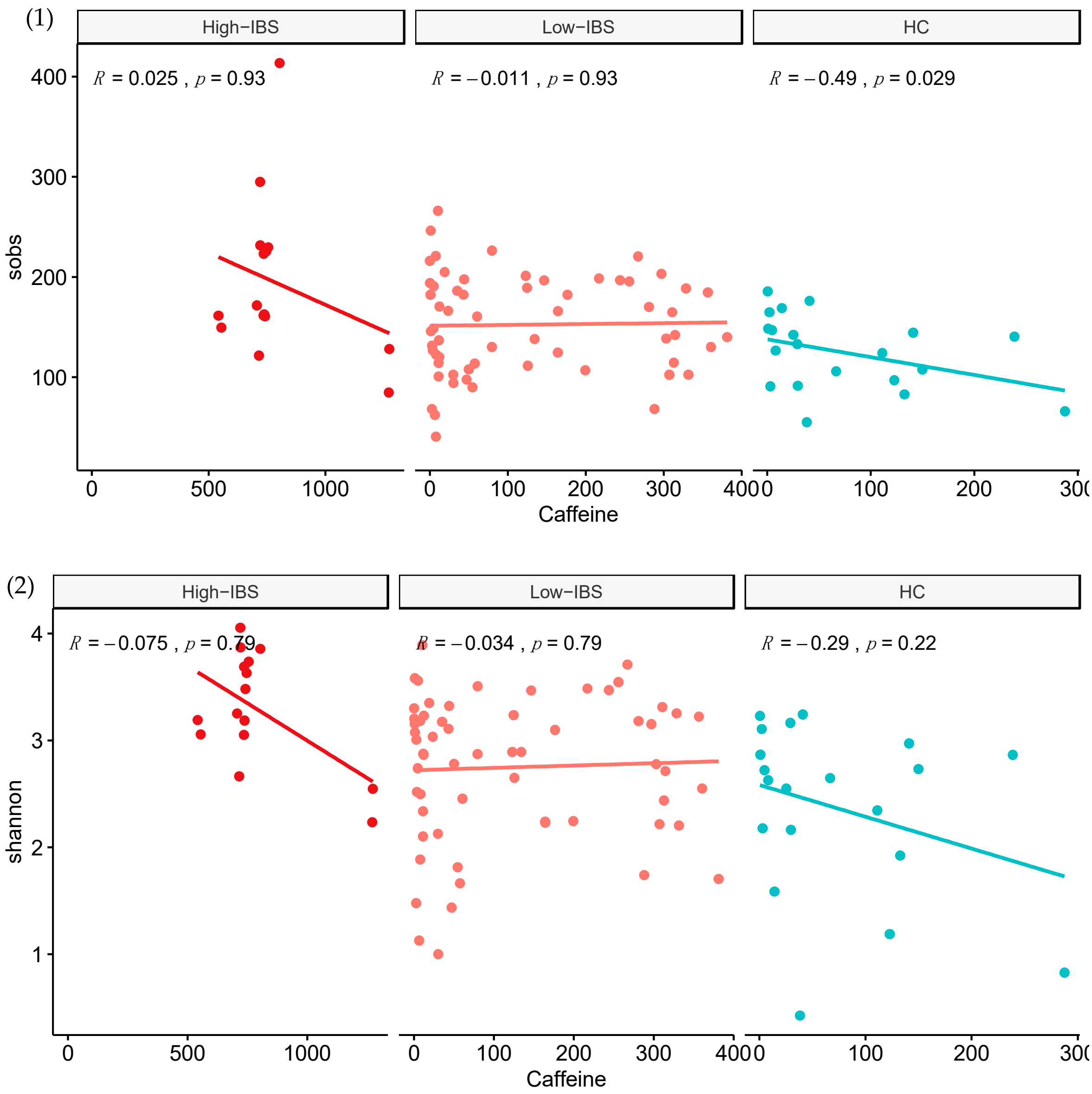

Figure S1. Correlation between caffeine intake and alpha diversity. (1): Correlation between total observed species (sobs) and caffeine intake. (2): Correlation between Shannon index and caffeine intake. High-IBS: Caffeine consumption more than 400 mg/day; Low-IBS: Caffeine consumption less than 400 mg/day.

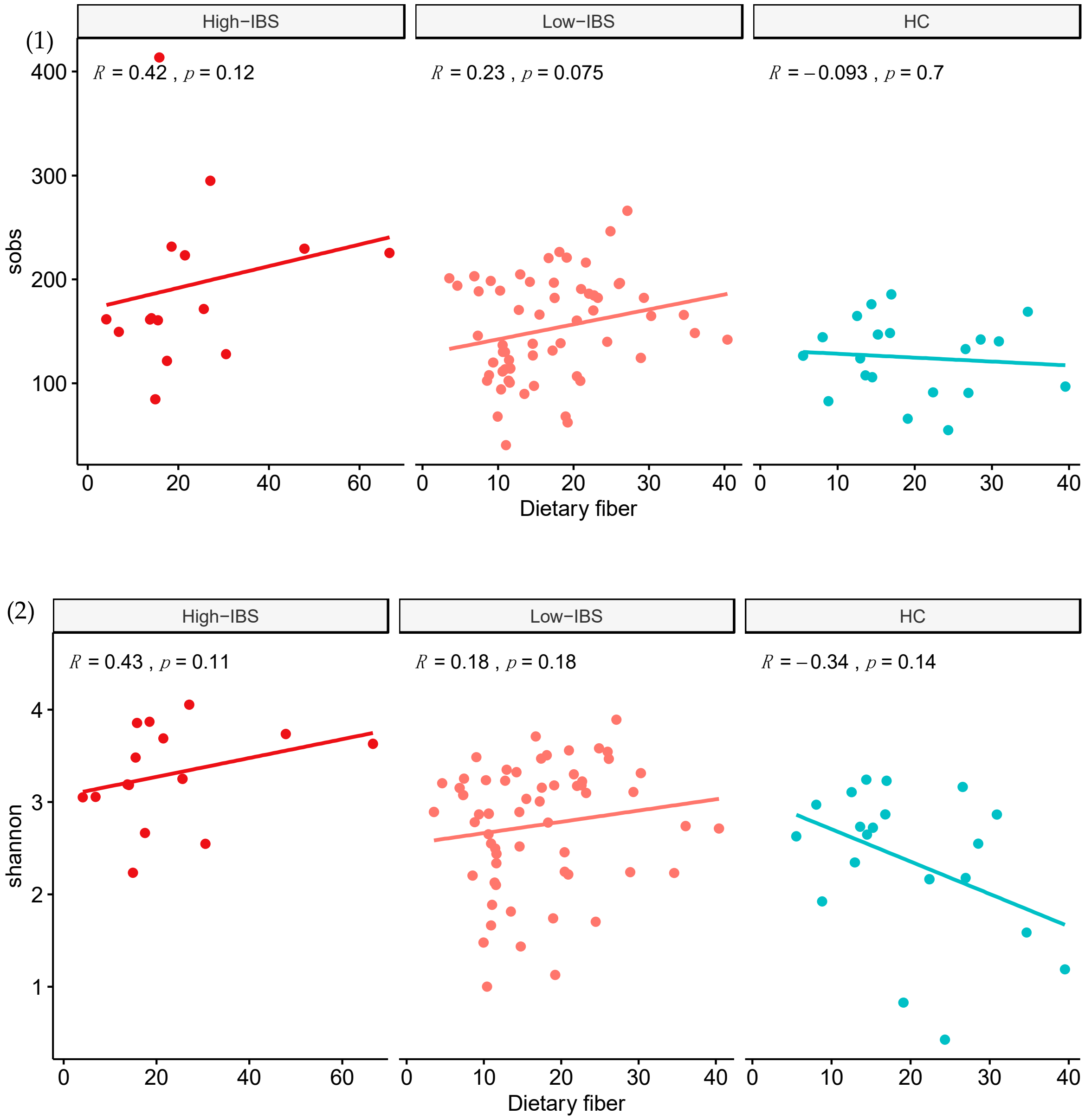

Figure S2. Correlation between dietary fiber intake and alpha diversity. (1): Correlation between total observed species (sobs) and dietary fiber intake. (2): Correlation between total Shannon index and dietary fiber intake. High-IBS: Caffeine consumption more than 400 mg/day; Low-IBS: Caffeine consumption less than 400 mg/day.

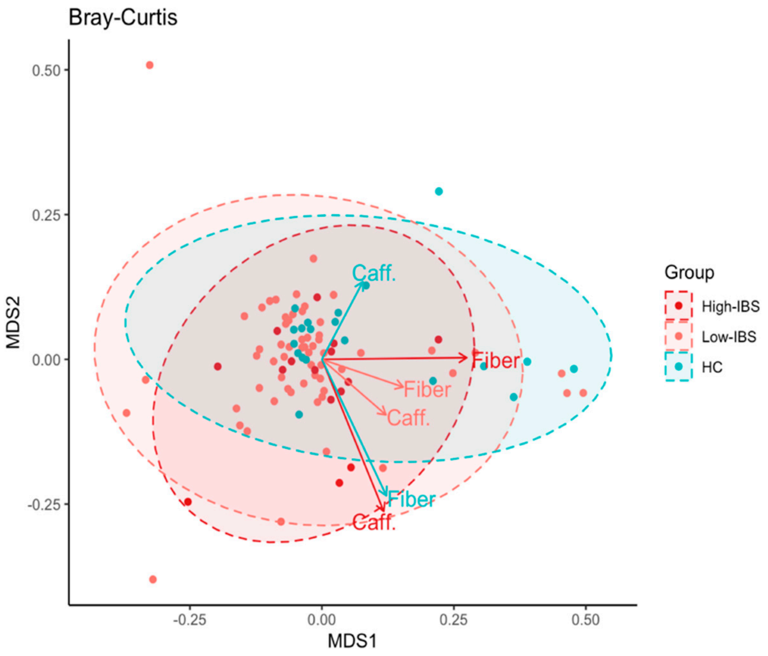

Figure S3. Correlation between beta diversity and nutrient intakes in High-IBS, Low-IBS and HC groups. High-IBS: Caffeine consumption more than 400 mg/day; Low-IBS: Caffeine consumption less than 400 mg/day; Caff: caffeine, Fiber: fiber
